# Mitochondria: hubs of cellular signaling, energetics and redox balance. A rich, vibrant, and diverse landscape of mitochondrial research

**DOI:** 10.3389/fphys.2015.00094

**Published:** 2015-03-26

**Authors:** Miguel A. Aon, Amadou K. S. Camara

**Affiliations:** ^1^Department of Medicine, School of Medicine, Johns Hopkins UniversityBaltimore, MD, USA; ^2^Department of Anesthesiology and Cardiovascular Research Center, Medical College of WisconsinMilwaukee, WI, USA

**Keywords:** energetic-redox compartmentation, posttranslational modification, apoptosis inducing factor, beta oxidation, permeability transition pore, infrared light heart protection, neuroprotection, mitochondrial network clustering

Mitochondria have become the cornerstone of cellular biology, opening new frontiers in health and disease. Poised at the convergence of most catabolic and anabolic pathways, mitochondria constitute the center of heterotrophic aerobic life, processing and generating key metabolites that feed into pathways leading to growth, division, and signaling. As such, mitochondria play the role of hubs in the overall metabolic network; thus their failure risks the collapse of most crucial cellular functions. The protagonist role of mitochondria is underscored by the existence of tightly regulated cellular processes that include autophagy/mitophagy and metabolically tuned morphological changes induced by fusion-fission dynamics. Mitochondria are also involved in a myriad of signaling cascades regulating cell survival vs. death. They provide the energy for the cell, a hub for biosynthetic processes and they contain a self-destructive arsenal of apoptotogenic factors that can be unleashed to promote apoptotic signaling. Consequently, it is no wonder that mitochondrial dysfunction is implicated in the aging phenomenon, and in numerous human maladies including metabolic disorders, cardiomyopathies, and neurodegeneration. Indeed, diseases characterized by altered gene-nutrient interactions, such as diabetes and cancer, exhibit elevated levels of reactive oxygen species (ROS) of which mitochondria are a major source. Therefore, mitochondrial dysfunction and the resulting oxidative stress are central in these and other human pathologies. Excess ROS induced by oxidative stress are specifically known to contribute to these pathogeneses in part by increasing mitochondrial DNA mutations that, via retrograde signaling, affects nuclear gene expression, ultimately modulating gene transcription, protein translation, post-translational modifications, and cellular signaling.

For this Research Topic, our initial focus was on the younger generation of researchers working on mitochondria, believing both in their responsibility to take the next steps in this field, confident in their ability to take mitochondrial research to new and exhilarating heights. In this endeavor, 18 papers, including a Commentary to one of the contributions, reveal an exciting, broad scope of subjects involving mitochondrial research that exploit a variety of novel methods, insights, and ideas. The potential implications of mitochondria at the crossroad of cellular injury and therapeutics are also addressed.

Fresh insights into actively investigated areas affecting mitochondrial/cellular redox balance are highlighted in a series of review articles, as applied to the etiology of heart disease and insulin resistance (Alleman et al., 2014), and the role of the renin angiotensin system (Vajapey et al., 2014). The role of cellular redox compartmentalization (matrix vs. extra-matrix) in leading mitochondrial function from normal to pathological conditions (Kembro et al., 2014), and the functional significance of the differential redox status exhibited by subcellular organelles (likely lysosomes, peroxisomes, endoplasmic reticulum, and nuclei) apart from mitochondria (Kaludercic et al., 2014) are also reported.

A review contribution by Birkedal et al. (2014), and an original research article by Kurz et al. (2014) highlight the use of new conceptual and computational tools to address intracellular energetic compartmentalization and mitochondrial network organization as essential components of the coordinated energetic response during the cardiac systole-diastole cycle. Hypertrophic cardiomyopathy, the most common inherited cardiac disease, is analyzed in a review by Vakrou and Abraham (2014) and defined as a metabolic disease in which mitochondrial function plays a relevant role.

A novel experiment on the protective role of infrared light against cardiac injury elicited under ischemia and reperfusion (IR) is presented in Keszler et al. (2014) and highlighted in a Commentary article by Karam and Akar (2014). In another study, Chen et al. (2014) report on the flavin-NADH-containing apoptosis-inducing factor (AIF), found in mitochondria and required for optimal respiratory function. AIF acts as a stimulator of ROS-mediated cell death during IR in the Harlequin mouse model that expresses AIF in reduced amounts. Additional studies using intact heart, analyze the role of mitochondrial ion channels in arrhythmic propensity under oxidative stress, during IR injury (Xie et al., 2014). Whereas low levels of ROS can serve as critical signaling molecules, excess ROS are implicated in IR injury. Lindsay et al. (2015) report on specific electron transport chain (ETC) complexes that are responsible for ROS generation under conditions that may prevail during prolonged ischemia. These include different substrate utilization, excessive mitochondrial calcium load and change in pH that culminate in mitochondrial permeability transition pore opening and ROS emission.

Signaling aspects related to mitochondrial activity also had a prominent contribution in this Research Topic. Protective strategies of mitochondrial energetic function in neurons based on Bcl-xL, a member of the anti-apoptotic Bcl-2 protein family, and directed to prevent cell death via mitochondrial permeability transition pore opening and outer membrane permeabilization, are addressed by Jonas et al. (2014). In a review article Papanicolaou et al. (2014) discussed the new emerging family of mitochondrial proteins that are post-translationally modified via direct reaction of lysine residues with activated thioester coenzyme A intermediates, and their functional impact on mitochondrial sirtuins. In a Perspective article, the role of mitochondrial dysfunction and the molecular mechanisms participating in necroptosis, a form of necrosis, are critically examined by Marshall and Baines (2014).

A relatively new area of research is covered by Dedkova and Blatter (2014) who extensively review the role of the ketone body β-hydroxybutyrate, its polymer poly-β-hydroxybutyrate, and inorganic polyphosphate, in diverse cellular functions, including mitochondrial ion transport, energetics and activation of mitochondrial permeability transition by polyphosphate.

Detailed mechanisms of potential cardioprotection by volatile anesthetics to target mitochondrial channels/transporters and ETC complexes are described in Agarwal et al. (2014). Recent emergent molecular processes mediating physical-metabolic interactions between lipid droplets and mitochondria and their potential impact on fatty acid oxidation and generation of signaling ROS are explored in the contribution by Aon et al. (2014).

Overall, the work in this Research Topic exemplifies many situations in which the morphological and functional behavior of mitochondria is sensitively tuned to the changing cellular energetic-redox status. The Research Topic sought clues about the underlying mechanisms that allow mitochondria to accomplish their energetic function while at the same time confronted with the unavoidable redox hurdle of processing huge amounts of oxygen as well as preserving the cellular redox environment. Since these conditions could be antithetical with the organelle's survival, many contributions shed light on the fact that mitochondrial targeted approaches to treat diseases could be a harbinger for their protection and concomitantly cytoprotection in different pathologies. In this case, we have come full circle: the protector, the mitochondrion, has to be protected.

## Conflict of interest statement

The authors declare that the research was conducted in the absence of any commercial or financial relationships that could be construed as a potential conflict of interest.
